# Physiological and Transcriptional Changes of Three Citrus Rootstock Seedlings under Iron Deficiency

**DOI:** 10.3389/fpls.2017.01104

**Published:** 2017-06-26

**Authors:** Lina Fu, Qingqing Zhu, Yinya Sun, Wei Du, Zhiyong Pan, Shu’ang Peng

**Affiliations:** ^1^Key Laboratory of Horticultural Plant Biology, Ministry of Education, Huazhong Agricultural UniversityWuhan, China; ^2^Key Laboratory of Horticultural Crop Biology and Genetic Improvement (Central Region), Ministry of AgricultureWuhan, China

**Keywords:** iron deficiency, citrus rootstocks, iron concentration, transcriptome analysis, gene expression regulation

## Abstract

Iron is an essential micronutrient for plants, and plants have evolved adaptive mechanisms to improve iron acquisition from soils. Grafting on iron deficiency-tolerant rootstock is an effective strategy to prevent iron deficiency-chlorosis in fruit-tree crops. To determine the mechanisms underlying iron uptake in iron deficiency, two iron deficiency-tolerant citrus rootstocks, Zhique (ZQ) and Xiangcheng (XC), as well as iron deficiency-sensitive rootstock trifoliate orange (TO) seedlings were studied. Plants were grown in hydroponics system for 100 days, having 50 μM iron (control) and 0 μM iron (iron deficiency) nutrient solution. Under iron deficiency, more obvious visual symptoms of iron chlorosis were observed in the leaves of TO, whereas slight symptoms were observed in ZQ and XC. This was further supported by the lower chlorophyll concentration in the leaves of TO than in leaves of ZQ and XC. Ferrous iron showed no differences among the three citrus rootstock roots, whereas ferrous iron was significantly higher in leaves of ZQ and XC than TO. The specific iron absorption rate and leaf iron proportion were significantly higher in ZQ and XC than in TO, suggesting the iron deficiency tolerance can be explained by increased iron uptake in roots of ZQ and XC, allowed by subsequent translocation to shoots. In transcriptome analysis, 29, 298, and 500 differentially expressed genes (DEGs) in response to iron deficiency were identified in ZQ, XC, and TO, respectively (Fold change ≥ 2 and Probability ≥ 0.8 were used as thresholds to identify DEGs). A Gene Ontology analysis suggested that several genotype-specific biological processes are involved in response to iron deficiency. Genes associated with cell wall biosynthesis, ethylene and abscisic acid signal transduction pathways were involved in iron deficiency responses in citrus rootstocks. The results of this study provide a basis for future analyses of the physiological and molecular mechanisms of the tolerance of different citrus rootstocks to iron deficiency.

## Introduction

Iron is an essential nutrient for plant growth since it takes part in most important primary metabolic processes, including photosynthesis, respiratory electron transport, nitrogen assimilation, and so on. Although the total iron content in soil is relatively high, its acquisition by crop plants is often limited owing to its low bioavailability in well-oxygenated and alkaline soil conditions (Mimmo et al., 2014). Chlorosis in leaves and decreased yield resulting from iron deficiency are serious problems for crop production.

Plants have developed two adaptive mechanisms under iron deficiency conditions: a reduction-based strategy I and a chelation-based Strategy II (Römheld and Marschner, 1986). In strategy I plants (dicotyledonous and non-grass monocotyledonous species), protons are secreted into the rhizosphere via plasma membrane-bound H^+^-ATPases in roots, which lowers the rhizosphere soil pH (Dell’Orto et al., 2000; Santi and Schmidt, 2009). Meanwhile ferric iron is reduced to the ferrous form by ferric chelate reductase (FCR) at the root surface (Robinson et al., 1999), and ferrous iron is then transported across root cell membranes through the iron-regulated transporter (IRT) (Eide et al., 1996). Strategy II plants (graminaceous species) secrete ferric-chelating substances, such as phytosiderophores, combine with ferric iron; the iron-siderophore complex is taken up by an oligopeptide transporter, Yellow stripe 1 (Curie et al., 2001). It is worth noting that most of these results have been obtained in model plants, and the adaptive mechanism of iron deficiency, is poorly understand in perennial woody plants.

Citrus is a commercially important fruit tree in the world, and most cultivars are susceptible to low iron bioavailability in calcareous soils (Carpena-Artes et al., 1995). Iron-fertilizer is usually used to supply adequate amounts of crop-available iron (Shenker and Chen, 2005; Rombolà and Tagliavini, 2006; Bacaicoa and García-Mina, 2009). However, iron deficiency chlorosis is difficult to correct owing to the rapid transformation of iron -fertilizers to an unavailable form in the soil (Fernández et al., 2004). Moreover, iron fertilizers not only increase costs, but also damage the environment. Beside iron–fertilizers, the microorganisms could increase bioavailability of the iron in soil, and they modulate iron acquisition in plants by an increased expression of typical genes related to iron deficiency response (Pii et al., 2015, 2016; Terrazas et al., 2016). Further studies are needed to better understand the interactions of plant-microorganism-soil for the acquisition of iron in calcareous soils.

Selecting iron deficiency-tolerant rootstocks is believed to be an effective and environment friendly strategy to minimize the iron cholorosis in the citrus industry (Ksouri et al., 2006, 2007; Molassiotis et al., 2006). Several recent studies have screened citrus rootstocks tolerant to iron deficiency based on physiological and morphological indexes (Pestana et al., 2005; Castle et al., 2009; Martínez-Cuenca et al., 2013). For example, compared with Carrizo citrange, iron deficiency-tolerant Cleopatra mandarin has a higher root ferric reduction rate and accumulates more iron in the root apoplast. Molecular responses have also been investigated by monitoring transcript expression in different citrus rootstocks. Genes involved in cell wall modification, photosynthesis, and oxidative stress are found significantly up-regulated under iron deficiency in the commonly used rootstock *Poncirus trifoliata* (L.) Raf (Forner-Giner et al., 2010). A set of genes associated with oxidative stress, hormone metabolism, and protein turnover are involved in iron deficiency acclimation by comparing iron deficiency-tolerant (Carrizo citrange) and iron deficiency-sensitive (Swinglecitrumelo) rootstocks, planted in calcareous soils (Licciardello et al., 2013).

In our previous study, we found that Zhique (ZQ), a novel citrus rootstock native to China, shows strong tolerance to iron deficiency in calcareous soil (Fu et al., 2016). Here, to further clarify the mechanism underlying the tolerance to iron deficiency in ZQ, an iron deficiency-sensitive rootstock trifoliate orange (TO) and a known iron deficiency-tolerant rootstock Xiangcheng (XC) were used as samples together, and the physiological and transcriptional responses of these three materials were investigated upon iron deficiency treatment.

## Materials and Methods

### Plant Culture

Seeds of Zhique (*Citrus wilsonii* Tanaka) were obtained from Chenggu Fruit Industry Technical Guidance Station, seeds of Xiangcheng (*C. junos Sieb*. ex Tanaka) were collected and provided by the Institute of Horticulture Research, Sichuan Provincial Academy of Agricultural Science, China, and seeds of TO [*P. trifoliate* (L.) Raf.] were obtained from the National Citrus Breeding Center in Huazhong Agricultural University, China. As described in our previous work (An et al., 2012), seeds of these three citrus rootstocks were surface-sterilized for 15 min in a 5% sodium hypochlorite solution. The seeds were placed on a porcelain tray with moistened gauze and transferred to an incubator at 30°C until germination. They were then cultivated in plastic pots filled with vermiculite. These seeds were grown in vermiculite until seedlings with four true leaves. Uniform size seedlings of each rootstock were selected, transferred to hydroponic culture medium containing 1/4 strength Hoagland’s nutrient solution (Hoagland and Arnon, 1950), and kept for about 3 weeks until new roots grew. Afterward, the seedlings were grown in modified Hoagland’s nutrient solution for 100 days containing either 50 μM iron-EDTA (control) or 0 μM iron-EDTA (iron deficiency). The pH was adjusted to 6.0 with NaOH for both control and iron deficiency treatments. The solution was ventilated for 20 min every 2 h and replaced once a week. The physiology experiment was kept until the symptom of iron deficiency was observed (about 100 days iron deficiency treatment). Moreover, to detect the iron response and expression of key genes in experimental rootstocks, root samples at a length of 2 cm from the apex were randomly collected at 0 h, 24, 48, 72, and 96 h of iron deficiency treatments.

### Measurement of Plant Growth Parameters

At the end of the experiment (100 days treatment), nine plants per treatment were harvested randomly and were rinsed with deionized water. Then the samples were divided into leaf, stem, and root samples. Leaf area (cm^2^) was determined using a leaf area meter (Li-3100C; LI-COR Biosciences Inc., Lincoln, NE, United States). The root samples were scanned using an Epson digital scanner (Expression 10000XL 1.0; Epson Inc., Nagano, Japan) and images were analyzed using WinRhizo Pro (S) version 2009c (Regent Instruments Inc., Quebec, Canada). The root traits examined included total root length (cm), root surface area (cm^2^), root volume (cm^3^) and root number. The fresh materials were placed into a forced air oven at 105°C for 15 min, and then at 75°C until constant weights were reached to determine dry weights (g). All dried samples were ground into a fine powder to determine iron concentration in various tissues. Seedling height (cm) and taproot length (cm) were measured using a measuring scale.

### Chlorophyll Content Determination

Chlorophyll content concentration was measured according to Fu et al. (2016). Chlorophyll concentration was calculated according to the following formulae:

$$\text{Chlorophylla }\left(\text{mg}{\text{L}}^{-1}\right)=12.7({\text{OD}}_{663})-2.69({\text{OD}}_{644});$$

$$\text{Chlorophyllb}\;(\text{mg}{\text{L}}^{-1})=22.9({\text{OD}}_{644})-4.68({\text{OD}}_{663});$$

$$\text{Chlorophyll content of the leaf }(\text{mg}{\text{m}}^{-1}\text{FW})\;=(\text{Chlorophyll concentration }\times \text{extraction solution volume }\times \text{dilution ratio})/\text{fresh weight.}$$

### Iron Concentration Determination

Total iron and ferrous iron concentrations in root, stem, and leaf samples were determined by different methods (Fu et al., 2016). The iron distribution is expressed as the iron content (concentration × dry weight) in one plant part relative to the total plant iron content. The ratio of the iron concentrations in different plant parts represents the ability of the plant to transport iron from the root to shoot, or the ability of the shoot to distribute iron to various shoot parts. Specific uptake rate of iron was calculated according to the equation of Bellaloui and Brown (1998) as follows:

Specific uptake rate (μg g^-1^d^-1^) = [(lnR_2_ - lnR_1_)/(T_2_ - T_1_)] × [(M_2_ - M_1_)/(R_2_ - R_1_)], where R_1_ and R_2_ are the initial and final root dry weights, respectively; T_2_ and T_1_are the treatment durations; M_1_ and M_2_ are the initial (T_1_) and final (T_2_) iron contents per plant.

### Transcript Analysis

For the transcript analysis, roots with length of 2 cm from the tips were randomly collected after 50 μM Fe-EDTA (+Fe) or 0 μM Fe-EDTA (-Fe) treatments for 24 h, and were immediately frozen in liquid nitrogen and stored at -80°C for further analysis.

Roots from nine plants were pooled as an independent biological replicate and three independent biological replicates were used in transcriptomic analysis. RNAs were extracted from ZQ, XC and TO roots and used to construct cDNA libraries, with three replicates for each treatment (6 for ZQ, 6 for XC, and 6 for TO). RNA library construction and sequencing experiments were conducted at Huada Genomics Co. Ltd. (Shenzhen, China). During the QC step, the Agilent 2100 Bioanalyzer (Santa Clara, CA, United States) and ABI StepOnePlus Real-Time PCR System were used for qualitative and quantitative characterization of the sample library. The library products were sequenced using the IlluminaHiSeq^TM^ 2000 (San Diego, CA, United States). The raw reads in fasta format were deposited in the NCBI Sequence Read Archive (SRA) database^1^ and the corresponding run accession number (SRR5272210) was offered.

As there are some adaptor sequences and/or low quality reads present in the raw reads, data filtering has been carried out by the SOAPnuke^2^ to obtain high quality reads as the clean reads (clean data). The procedure included following steps: (1) Remove reads with adaptor sequences; (2) Remove reads in which the percentage of unknown bases (N) is greater than 10%; (3) Remove low quality reads. If the percentage of the low quality base (base with quality value ≤ 5) is greater than 50% in a read, we define this read as low quality. Subsequently, the proportion of clean reads in raw reads of the libraries was classified. The clean reads were mapped to the sweet orange genome^3^ (Xu et al., 2012) using SOAP (Li et al., 2009), and the NCBI database^4^. Mismatches at no more than 2 bases were allowed in the alignment. The reads mapped to reference sequences from multiple genes were filtered. Subsequently, a sequencing saturation analysis and randomness assessments were carried out (**Supplementary Figures S2–S4**). RSEM is a quantification tool that computed Maximum likelihood abundance estimates using the Expectation Maximization (EM) algorithm for its statistical model, including the modeling of paired-end (PE) and variable-length reads, fragment length distributions, and quality scores, to determine which transcripts are isoforms of the same gene (Li and Dewey, 2011).

FPKM method is used in calculating expression level, the formula is following: FPKM = 10^6^C/(NL/10^3^). Here FPKM (A) is the expression level of gene A, *C* is number of reads that uniquely aligned to gene A, *N* is total number of reads that uniquely aligned to all genes, and *L* is number of bases of gene A. The FPKM method is able to eliminate the influence of different gene length and sequencing discrepancy on the calculation of gene expression level. Therefore, the FPKM values can be directly used for comparing the difference of gene expression among samples.

If there is more than one transcript for a gene, the longest one is used to calculate its expression level and coverage. A fold change ≥ 2 and Probability ≥ 0.8 were used as thresholds to identify DEGs. Gene Ontology (GO) annotation was performed by using Blast2GO software (GO association done by a BLASTX against the NCBI nr database). For the pathway enrichment analysis, all DEGs were mapped to pathway terms in the KEGG database^5^. Main functional categories for differentially expressed genes were analyzed according to MapMan functional plant categories (Lohse et al., 2014).

### Quantitative Real-Time PCR Analysis

The detailed protocol for the quantitative real-time PCR analysis procedure was described previously (An et al., 2012). Briefly, total RNA was isolated with TRIzol reagent (RNAiso Plus; Takara, Shiga, Japan) according to the protocol provided by the manufacturer. RNA quality (OD_260_/OD_280_ ratio) and concentration were measured. Based on the estimated RNA concentration, one microgram of total RNA was used per cDNA synthesis reaction to avoid potential bias in the transcript evaluation. The actin gene was used for normalization. The primer sequences are listed in Supplementary Table S1. Quantitative real-time PCRs reactions were performed with SYBR Green PCR Master Mix (SYBR Premix Ex Taq II; Takara) and analyzed using the Real-Time System. Reactions were initiated with an initial incubation at 50°C for 2 min and 95°C for 10 min, followed by 40 cycles of 95°C for 15 s, and 60°C for 30 s. Four technical replicates were assayed for all PCRs. The Livak method (Livak and Schmittgen, 2001) was employed to calculate the relative expression levels.

### Statistical Analysis

All experiments data were tested for differences using analysis of variance, and mean separation within samples by Duncan’s multiple range test (SAS version 8.1; SAS Institute, Cary, NC, United States).

## Results

### Morphological and Growth Parameters of ZQ and XC Differed from Those of TO

**FIGURE 1:**
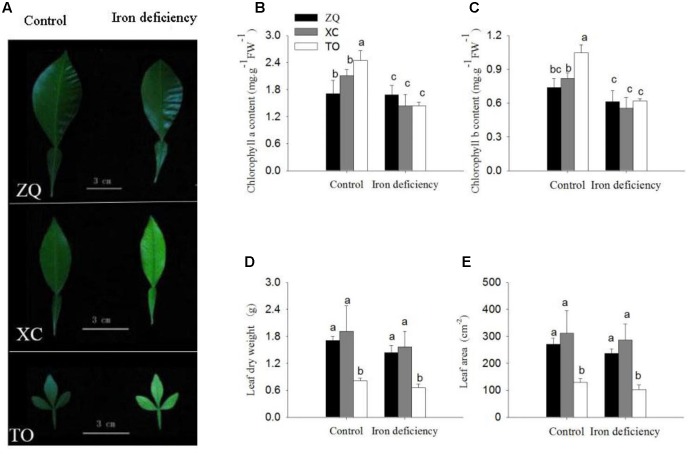
Difference in leaf growth parameters of Zhique (ZQ), Xiangcheng (XC), and trifoliate orange (TO) grown in control and iron deficiency nutrient solution for 100 days. **(A)** Leaf morphology in ZQ, XC, and TO. **(B,C)** Chlorophyll concentration of leaves in ZQ, XC, and TO. **(D,E)** Leaf dry weight and leaf area of leaves in ZQ, XC, and TO. Letters (a, b, and c) indicate significant differences within samples via Duncan’s multiple range test *P* < 0.05 (Means ± SEM, *n* = 3).

Three citrus rootstock seedlings, ZQ (Zhique, tolerant to iron deficiency), XC (Xiangcheng, tolerant to iron deficiency) and TO (trifoliate orange, sensitive to iron deficiency), were grown in hydroponic culture and subjected to iron deficiency. After 100 days of iron deficiency, the young leaves of TO showed obvious interveinal chlorosis under iron deficiency conditions. By contrast, the ZQ and XC leaves showed only slight chlorosis under iron deficiency treatments (**Figure 1A**). Consistent with the observed phenotypes, the reduction in chlorophyll a and b content was greater in TO (41 and 41%) than in ZQ (1 and 16%) and XC (31 and 32%) under iron deficiency conditions (**Figures 1B,C**). The symptoms of iron deficiency and the reductions in chlorophyll a and b content were also obviously greater in XC than in ZQ. However, no differences were detected in leaf dry weight and leaf area among the three rootstocks under iron deficiency (**Figures 1D,E**).

The stem morphologies are shown in **Supplementary Figure S1**. No significant differences were found in stem length and stem dry weight among ZQ, XC, and TO.

**FIGURE 2:**
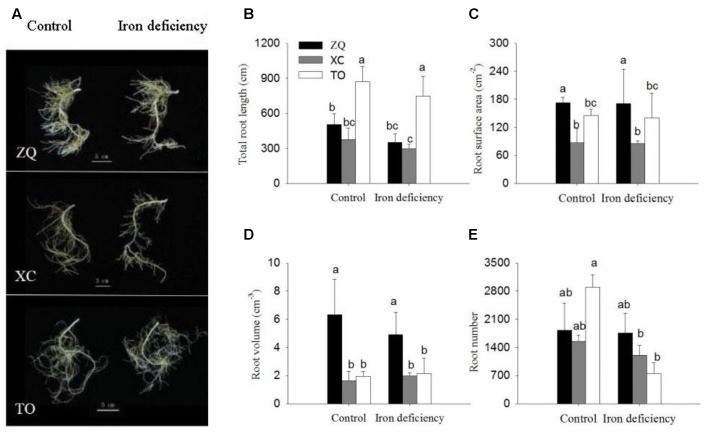
Root growth parameters of ZQ, XC, and TO grown in control and iron deficiency nutrient solution for 100 days. **(A)** Root morphology in ZQ, XC, and TO. **(B–E)** Root growth parameters in ZQ, XC and TO. Letters (a, b, and c) indicate significant differences within samples via Duncan’s multiple range test *P* < 0.05 (Means ± SEM, *n* = 3).

As shown in **Figure 2**, the roots morphology of the three rootstock seedlings exhibited no obvious differences under iron deficiency treatment as compared with control roots. However, under iron deficiency conditions, the root number were significantly reduced in TO, while no difference was detected in ZQ and XC root growth parameters under iron deficiency treatment as compared with control roots, including total root length, root surface area, root volume, and root numbers (**Figure 2**).

### Specific Fe Absorption Rate Was Higher in Roots of ZQ and XC than in Roots of TO

**FIGURE 3:**
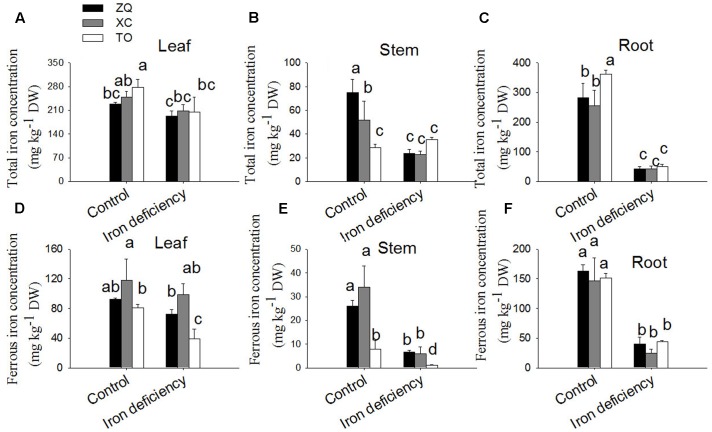
Total Fe and ferrous Fe concentrations in ZQ, XC, and TO grown in control and iron deficiency nutrient solution for 100 days. **(A–C)** Total iron concentrations in ZQ, XC, and TO. **(D–F)** Ferrous iron concentrations in ZQ, XC, and TO. Data are shown as means ± SEM (*n* = 3). Letters (a, b, and c) indicate significant differences within samples via Duncan’s multiple range test *P* < 0.05 (Means ± SEM, *n* = 3).

Total and ferrous iron concentrations in different tissues were determined to explore iron uptake and allocation in citrus seedlings. As shown in **Figures 3A,D**, the total and ferrous iron in ZQ and XC showed no differences in leaves from those of control plant leaves under iron deficiency treatments. However, in leaves of TO both total and ferrous iron were significantly lower than those of control plant leaves. In stems under iron deficiency treatments total and ferrous iron were significantly decreased in ZQ and XC (**Figures 3B,E**). In roots, total and ferrous iron level in all the three rootstocks were significantly decreased under iron deficiency conditions (**Figures 3C,F**).

**FIGURE 4:**
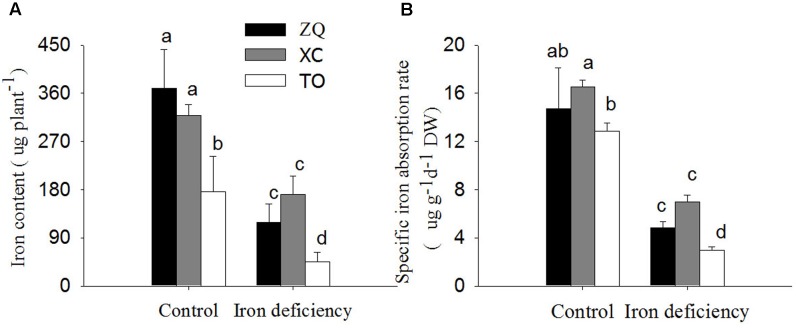
Fe content per plant and specific Fe absorption rate in ZQ, XC, and TO grown in control and iron deficiency nutrient solution for 100 days. **(A)** Fe content per plant in ZQ, XC, and TO. **(B)** Specific Fe absorption rate in ZQ, XC, and TO. Letters (a, b, and c) indicate significant differences within samples via Duncan’s multiple range test *P* < 0.05 (Means ± SEM, *n* = 3).

**FIGURE 5:**
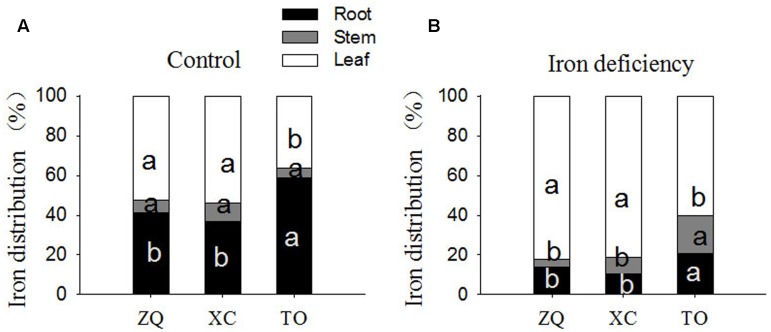
Fe distribution in roots, stems and leaves of ZQ, XC, and TO grown in control and iron deficiency nutrient solution for 100 days. **(A)** Fe distribution in roots, stems, and leaves of ZQ, XC, and TO under control. **(B)** Fe distribution in roots, stems and leaves of ZQ, XC, and TO under iron deficiency. Letters (a, b, and c) indicate significant differences within samples via Duncan’s multiple range test *P* < 0.05 (Means ± SEM, *n* = 3).

Iron content per plant and specific iron absorption rate in all the three genotypes were significantly lower under iron deficiency conditions than in control seedlings (**Figure 4**). It is worth noting that the specific iron absorption rate was higher in ZQ and XC than in TO under iron deficiency (though the difference between ZQ and TO was not rich a significant), but no difference among the three rootstocks were detected under control conditions (control). With respect to the iron distribution (**Figure 5**), iron deficiency treatment induced an increase in the leaf iron proportion. Notably, the leaf iron proportions in ZQ and XC were significantly higher than those in TO, regardless of iron treatments. The opposite trend was observed for relative root iron proportion.

### Transcriptome Changes in Response to Iron Deficiency

To investigate transcriptomic changes happened after 24 h iron deficiency, RNA was extracted from ZQ, XC, and TO roots and used to construct cDNA libraries. More than 12 million total reads per library were obtained and greater than 99.59% of total reads were identified as clean reads (Supplementary Table S2). Based on a sequence saturation analysis, the number of detected genes was saturated and the sequencing depth was sufficient for gene expression analysis (data not shown). The total clean reads were then mapped to the sweet orange genome^6^. A total of 24,140 transcripts were detected (Supplementary Tables S2, S3). The expression level of each gene was normalized and evaluated by the FPKM approach (Supplementary Table S4); detailed expression and annotation information is provided in Supplementary Table S3. DEGs were defined (Supplementary Table S5). Functional analyses of DEGs were performed, including KEGG pathways (Supplementary Table S6) and a GO analysis (Supplementary Table S7).

**FIGURE 6:**
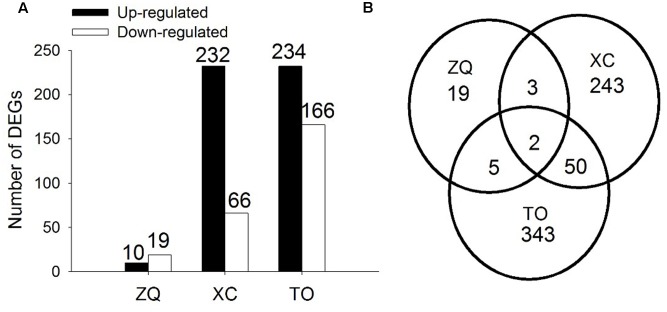
Differentially Expressed Genes in roots of citrus rootstocks in 24 h of control and iron deficiency conditions. **(A)** Numbers of differentially expressed genes. **(B)** Venn diagram showing the number of genes that are differentially expressed in ZQ, XC, and TO, including shared and independent genes.

**FIGURE 7:**
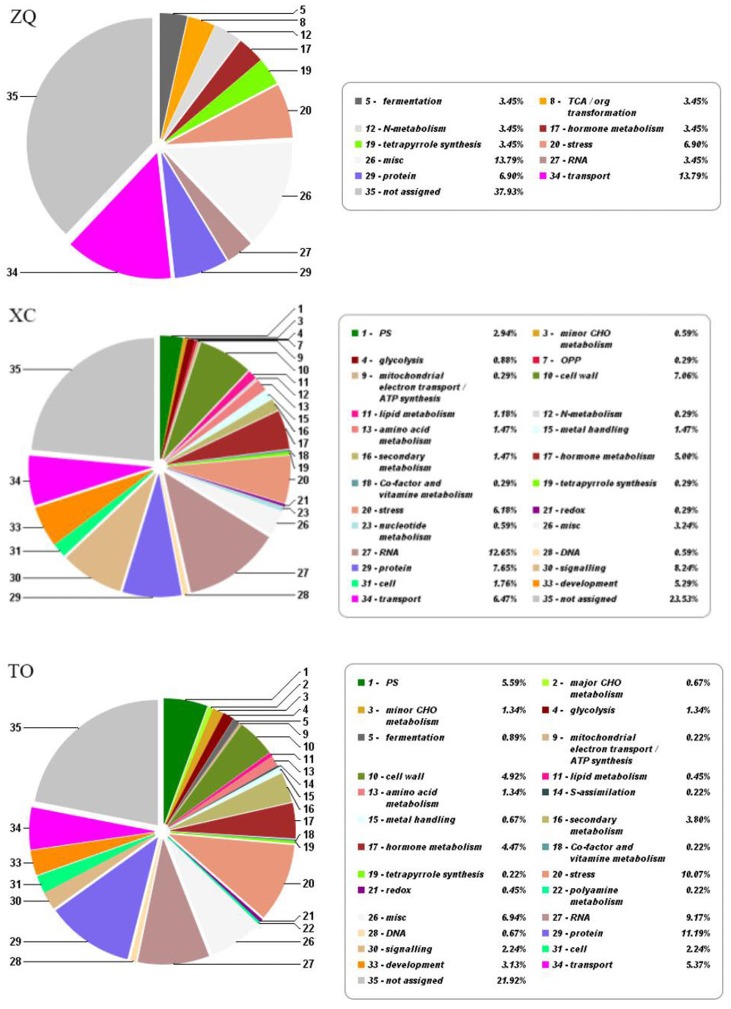
Main functional categories for differentially expressed genes according to MapMan functional plant categories.

In ZQ, only 29 genes were differentially expressed in response to iron deficiency, including 10 up-regulated and 19 down-regulated genes. In XC, 298 genes were differentially expressed, including 232 up-regulated and 66 down-regulated genes. In TO, 400 genes were differentially expressed, including 234 up-regulated and 166 down-regulated genes (**Figure 6**). Among these DEGs, only two (Cs4g18450 and Orange1.1t00580) were common to the three rootstocks. Five DEGs were common to both ZQ and XC, 7 DEGs were common to both ZQ and TO, and 52 DEGs were common to both XC and TO. Functional categories of the DEGs are summarized in **Figure 7**. The major categories were “transport” (13.79%),“protein” (6.90%), and “stress” (6.90%) in ZQ, “RNA” (12.65%), “signaling” (8.24%), and “protein” (7.65%) in XC, and “protein” (11.19%), “stress” (10.07%), and “RNA” (9.17%) in TO. In addition, genes involved in “hormone metabolism,” “cell,” and “secondary metabolism” were also highly represented in the three rootstocks. To validate the RNA-seq results, expression levels of 20 DEGs in ZQ, XC, and TO were determined by real-time PCR. As expected, 17 out of 20 genes in ZQ, 19 out of 20 in XC, and 16 out of 20 in TO showed the same expression as detected by RNA-seq (**Supplementary Figure S5**), suggesting that the transcriptomic data is reliable.

The conservation of the mechanisms underlying the response to iron deficiency among citrus and other plant species were examined. The DEGs identified in ZQ, XC, and TO were compared with iron deficiency-related transcriptome data obtained for other species, including *Malus xiaojinensis*, *Poncirus trifoliate*, *Medicago truncatula*, and *Arabidopsis thaliana* (O’Rourke et al., 2009; Yang et al., 2010; Rodríguez-Celma et al., 2013a,b; Wang et al., 2014). The DEGs of the plant species under iron deficiency were blasted against the *Arabidopsis* whole genome sequences^7^ to search for homologs with an*E*-value = 1 × e^-5^ or lower. This approach yielded 17 genes responsive to iron deficiency in ZQ, XC, TO, *Malus xiaojinensis*, *Poncirus trifoliate*, *Medicago truncatula*, and *Arabidopsis thaliana* (**Table 1**). The genes were associated with hormone synthesis and signaling, transcription, transportation, cell wall modification, and degradation, indicating that these genes or biological processes might be conserved in iron homeostasis in both woody and herbaceous plants.

**Table 1:** A subset of differentially expressed genes conserved in citrus and several other species under iron deficiency.

Citrus gene ID	Gene annotation	ZQ(log_2_)	XC(log_2_)	TO(log_2_)	*Malus xiaojinensis*	*Poncirus trifoliata*	*Medicago truncatula*	*Arabidopsis thaliana*
Cs3g19420	Ethylene-responsive transcription factor 012	-1.46	**2.91**	**2.54**		√		
Cs9g13610	Ethylene-responsive transcription factor 105	-0.10	**2.44**	**1.11**	√			
Cs4g12540	Ethylene synthesis/degradation	0.57	-**6.25**	-**5.57**				√
Cs3g26100	Gibberellin-regulated protein 1	0.38	**1.18**	**1.03**			√	√
Cs5g03420	BTB/POZ domain-containing protein	-0.01	**2.82**	**1.00**			√	
Cs2g03240	Protein TIFY 5A	-0.92	**2.47**	**1.50**			√	
Cs4g10930	Late embryogenesis abundant protein Lea14-A	-0.09	**1.63**	-**1.04**			√	
Cs5g10740	Geranylgeranyl diphosphate Reductase	-0.21	**1.58**	0.99				
Cs7g21230	Exostosin-like 3	-0.05	**1.30**	**1.19**			√	
orange1.1t00580	Myb-like protein G	-**1.88**	-**1.69**	-**2.32**			√	
Cs6g19940	Na^+^ dependent neutral amino acid transporter	-0.65	-**1.78**	-**1.48**			√	
Cs3g15140	Nicotianamine synthase	-0.97	-**2.28**	-**1.19**				√
Cs2g28870	Peptide transporter PTR1	-0.50	-**2.62**	-**1.52**				√
Cs8g12690	Phosphoenolpyruvate carboxylase kinase 1	0.05	-**2.74**	-**1.19**			√	√
Cs4g03210	Xyloglucan endotransglucosylase protein	-1.77	**3.24**	**2.31**			√	
Cs2g02310	Uncharacterized protein	-0.20	-**2.77**	-**2.12**				√
Cs9g02930	Flavone synthase	1.24	-**6.28**	-**5.83**				√

*Data in bold represent differentially expressed genes*.

### Iron Related Genes Specifically Expressed under Different Iron Deficiency Period

**FIGURE 8:**
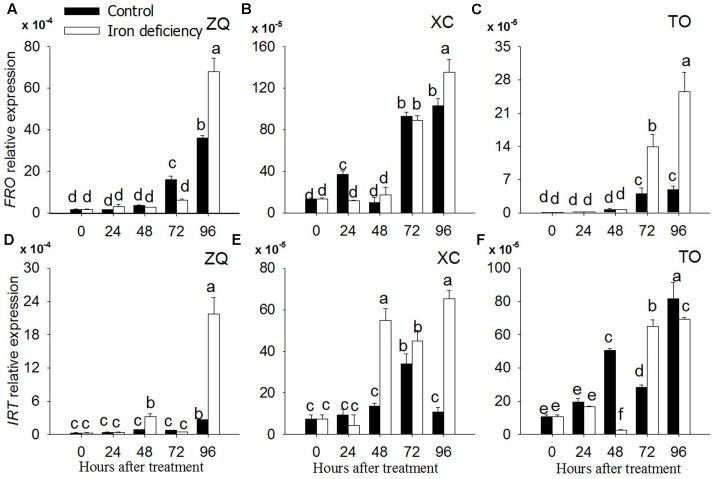
Gene expression of FRO and IRT in ZQ, XC and TO under iron deficiency for 0, 24, 48, 72, and 96 h. **(A,D)** The expression of FRO and IRT genes in ZQ. **(B,E)** The expression of FRO and IRT genes in XC. **(C,F)** The expression of FRO and IRT genes in TO.

Iron related genes were identified by a homology search with genes from sweet orange genome (Xu et al., 2012). Iron related genes such as *FRO* gene coding the plasma membrane ferric chelate reductase enzymes, and *IRT* gene coding for the iron transporter (**Figure 8**), were analyzed by quantitative real-time PCR at 0, 24h, 48, 72, and 96 h of iron deficiency treatments.

The expression of the *FRO* gene did not show any significant changes with regard to 24, 48, and 72 h iron deficiency in roots of ZQ and XC whereas *FRO* expression in both rootstocks increased significantly at 96 h iron deficiency. The *FRO* expression in TO was same to ZQ and XC at 24 and 48 h iron deficiency, whereas it was up-regulated at both 72 and 96 h iron deficiency in TO.

Iron-regulated transporter expression in ZQ and XC showed significantly up-regulated at 48, 72, and 96 h iron deficiency. However, the expression of IRT in TO was not significantly changed during the iron deficiency treatments.

## Discussion

### Physiological Mechanism of Tolerance to Iron Deficiency in Citrus Rootstocks

Zhique and XC are more tolerant to iron deficiency than TO (Li et al., 2001; Fu et al., 2016), consistent with our results showing that the ZQ and XC had better physiological performance under iron deficiency. Generally, the leaves were highly affected by iron deficiency in TO, as evidenced by observed chlorosis, whereas minor effects on the growth of stems and roots were detected. Accordingly, the reduction rate of chlorophyll a and b contents were higher in TO than in ZQ and XC. Interestingly, they might be different tolerant mechanism to iron deficiency as ZQ and XC have very different levels of iron deficiency chlorosis. Considering the important role of iron in chlorophyll synthesis and chloroplast structure formation (Marschner and Römheld, 1995; Soldatini et al., 2000; M’sehli et al., 2009), our results indicated that iron contents or activities might be different among the leaves of the three rootstock seedlings.

As predicted, under iron deficiency treatment, the total iron and ferrous iron concentrations in leaves were significantly decreased in TO, but not in ZQ and XC. It is interesting that, under iron deficiency, though the total iron concentrations did not differ among ZQ, XC, and TO, the ferrous iron concentration iron in ZQ and XC were much higher than that in TO. These results supported a previous hypothesis that the ferrous concentration is an important indicator of iron deficiency-tolerance (Dasgan et al., 2003; Ksouri et al., 2006; Jelali et al., 2011; Fu et al., 2016), which could be further used to diagnose iron-deficiency symptom in citrus cultivation management. It is worth mentioning that both the total and ferrous iron in roots showed no differences among the three rootstocks, different from our previous results showing that the ferrous iron concentration in roots of ZQ was significantly higher than that in the roots of TO. This difference might be explained by (i) a difference in sample origin (the previous samples were collected in field conditions while samples from this study are from hydroponic culture), and (ii) a possible scion-rootstock combination effect in the previous study (mandarin/rootstock grafted trees), while rootstock seedlings were analyzed in this study.

A decrease in the ferrous iron concentration in leaves might be a consequence of lower iron uptake by roots and/or impaired iron translocation from roots to shoots. To clarify this, the iron content per plant, specific iron absorption rate, and iron distribution in different tissues of three rootstocks were investigated. Both the iron content per plant and specific iron absorption rate in iron deficiency-tolerant ZQ and XC were higher than those in iron deficiency sensitive TO under iron deficiency. Consistent with our results, Martínez-Cuenca et al. (2013) observed a greater increase in the iron uptake rate in the tolerant rootstock Cleopatra mandarin than in the iron deficiency-sensitive Carrizo citrange. These results indicate that, under iron deficiency, the tolerant rootstocks are more efficient in iron uptake by roots. In addition, our results showed that iron deficiency promotes the increase in the leaf iron distribution in all three rootstocks, but the leaf iron proportion in ZQ and XC were higher than those in TO. Together, our results indicated that tolerance to iron deficiency might be explained by increased iron uptake in the roots of ZQ and XC, and subsequently, increased translocation of iron from roots to shoots.

### Genes Associated with Iron Deficiency Acclimation in Citrus Rootstock

A comparative transcriptome analysis was performed to investigate the molecular mechanism underlying iron uptake in the roots of ZQ, XC, and TO under iron deficiency. More DEGs were detected in iron deficiency-sensitive TO than in ZQ and XC (400 genes against 298 and 29 genes, respectively). These results imply that the degree of molecular response could be arranged as TO > XC > ZQ, contrary to the performance of three rootstocks in filed conditions, in which ZQ is the most tolerant to iron deficiency while TO is the most sensitive.

Well-known iron-related genes, such as *FRO* and *IRT* genes, were not detected in our transcriptome analysis. One DEG was annotated as iron transport protein 3 (Cs4g18450), but it was down-regulated by iron deficiency treatment in all three rootstocks. Iron transporters, such as *IRT1* are up-regulated in other species, in transcriptome experiments regardless of growth conditions and duration of iron deficiency (Eide et al., 1996). The down-regulation of iron transport protein 3 in this study suggests that: (i) it may not be involved in iron uptake or (ii) it is an iron efflux gene, and its down-regulation under iron deficiency could prevent iron excretion and thus maintain more iron in root cells.

We infer that the absent of well-known iron-related genes such as *FRO* and *IRT* in DEG list may due to that the time (24 h) for iron deficiency treatment is not long enough, which was further confirmed by our quantitative PCR results. It was suggested that, different from herbaceous plants, the iron may be stored in apoplast which could be available for normal growth in woody plants (Martínez-Cuenca et al., 2013). However, after 24 h treatment, the expression levels of *IRT* gene in ZQ and XC were significantly up-regulated as compared with control, while it was relative stable in TO. As for *FRO*, its expression levels were similar in the three citrus rootstocks. The results may indicate that the expression of *IRT* rather than *FRO* is related with the higher iron uptake rate in ZQ and XC.

In total, 16 DEGs in XC and TO be involved in cell wall metabolism. Among them, 5 xyloglucan endotransglycosylase/hydrolase (XTH) genes in XC and TO were up-regulated under iron deficiency. XTH plays roles in cell wall modification by regulating cell wall strength and extensibility, and is involved in diverse environmental stress responses (Herbers et al., 2001; Wu et al., 2005; Cho et al., 2006). Notably, the expression level of all five XTH genes in XC were at least four times higher than those in TO, suggesting that the cell wall modification in iron deficiency was more intense in XC than in TO. In addition, one pectin lyase gene in XC and TO was down-regulated, suggesting that the roots provide more pectin for cell wall restructuring by inhibiting pectin degradation. Compared with TO, the expression level of the pectin lyase gene was much lower in XC. Forner-Giner et al. (2010) found that the cell wall becomes thinner in iron deficiency in TO, and this may result from cell wall restructuring in response to iron deficiency. Therefore, the results of our study implied that the up regulation of xyloglucan endotransglycosylase/hydrolase and the down regulation of pectin lyase may play important roles in response to iron deficiency by enhancing cell wall modifications, especially in tolerant rootstocks.

One gene in ZQ (down-regulated), 16 genes in XC (15 up-regulated and 1 down-regulated), and 10 genes in TO (6 up-regulated and 4 down-regulated) involved in ethylene metabolism were detected in our study. A total of 13 genes involved in ethylene metabolism increased more than twofold in XC under iron deficiency, and only one gene in TO show an increase of twofold. In total, 13 up-regulated genes in XC encoded ethylene-responsive transcription factors (ERF) involved in the ethylene signal transduction pathway. Consistent with our results, several ERF genes are also activated in response to early iron deficiency in iron deficiency-tolerant *Malus xiaojinensis*, suggesting that the ethylene signal pathway plays an important role in iron-tolerant species under iron deficiency conditions. Another ethylene transcription factor family including ETHYLENE INSENSITIVE3 (EIN3) and ETHYLENE INSENSITIVE3-LIKE1 (EIL1), physically interact with FIT and activate iron acquisition-related genes under iron deficiency conditions (Lingam et al., 2011). Moreover, ERF functions downstream of EIN3, which may regulate one branch of the ethylene response pathway (Wang et al., 2002). These studies imply that the ERF genes detected here may be involved in the up-stream regulation of the response to iron deficiency in iron deficiency-tolerant citrus rootstocks.

It is notable that eight genes involved in the abscisic acid (ABA) pathway, an early response to iron deficiency, were detected in TO, including 2 up-regulated and 6 down-regulated genes, but genes in this pathway were not detected in ZQ and XC. Cs6g19380 (ABA 8′-hydroxylase) involved in ABA catabolism (Okamoto et al., 2006), was up-regulated and the Cs5g14370 (9-*cis* epoxycarotenoid dioxygenase, NCED), a key enzyme in the biosynthesis of ABA (Tan et al., 2003), was down-regulated. These data suggested that ABA biosynthesis was inhibited and the degradation process was activated in roots of TO under iron deficiency. In addition, an ABA-insensitive-like protein (cs3g23480) involved in ABA signal transduction (Gao et al., 2016) was also down-regulated in the roots of TO. Together, the iron deficiency-induced ABA metabolism response was only detected in the iron deficiency-sensitive rootstock TO and not in ZQ or XC, strengthening the idea that ZQ and XC are indeed more tolerant than TO, particularly considering that ABA is a stress response hormone.

## Conclusion

Our study revealed two new findings: (i) iron deficiency-tolerance in citrus rootstocks could be due to a higher uptake rate of iron and increased iron translocation from roots to shoots, and (ii) the cell wall modification, ethylene, and ABA signaling pathways seemed are involved in the acclimation to iron deficiency in citrus rootstocks. This study investigated the physiological and molecular changes underlying tolerance to iron deficiency, and it will contribute to further understand the tolerance response in citrus plants.

## Author Contributions

LF, ZP, and SP conceived the design of this research. LF and QZ performed the bioinformatics data analysis. LF wrote the paper. ZP contributed to the statistical analyses and in the revising of the manuscript. QZ, YS, and WD contributed to the revising of the manuscript.

## Conflict of Interest Statement

The authors declare that the research was conducted in the absence of any commercial or financial relationships that could be construed as a potential conflict of interest.

## Footnotes

1 https://www.ncbi.nlm.nih.gov/Traces/sra_sub/

2 http://soap.genomics.org.cn/

3 http://citrus.hzau.edu.cn/orange/index.php

4 http://www.ncbi.nlm.nih.gov/

5 http://www.genome.jp/kegg/

6 http://citrus.hzau.edu.cn/orange/index.php

7 http://www.arabidopsis.org/servlets/TairObject?type=gene\&id=137982

## Supplementary Material

FIGURE S1 Stem growth parameters of Zhique (ZQ), Xiangcheng (XC), and trifoliate orange (TO) grown in control and iron deficiency nutrient solution for 100 days. **(A)** Stem morphology in ZQ, XC, and TO. **(B)** Stem length in ZQ, XC, and TO. **(C)** Stem dry weight in ZQ, XC, and TO. Letters (a, b, and c) indicate significant differences within samples via Duncan’s multiple range test *P* < 0.05 (Means ± SEM, *n* = 3).

FIGURE S2 Curve of sequencing saturation in ZQ. *X*-axis shows the number of clean reads, units is 100 k – extreme value is currently the volume of sequencing. *Y*-axis shows the ratio of identified gene number to number of total gene reported in database.

FIGURE S3 Curve of sequencing saturation in ZQ. *X*-axis shows the number of clean reads, units is 100 k – extreme value is currently the volume of sequencing. *Y*-axis shows the ratio of identified gene number to number of total gene reported in database.

FIGURE S4 Curve of sequencing saturation in ZQ. *X*-axis shows the number of clean reads, units is 100 k – extreme value is currently the volume of sequencing. *Y*-axis shows the ratio of identified gene number to number of total gene reported in database.

FIGURE S5 Log_2_ transcription levels under iron deficiency relative to control levels estimated by qRT-PCR and by RNA-seq for ZQ, XC, and TO after 24 h of iron deficiency.
