# Supplementary figures and images for: Physiological and Transcriptional Changes of Three Citrus Rootstock Seedlings under Iron Deficiency
Source: Front Plant Sci. 2017 Jun 26;8:1104. doi: 10.3389/fpls.2017.01104 (PMC5483480; doi:10.3389/fpls.2017.01104)

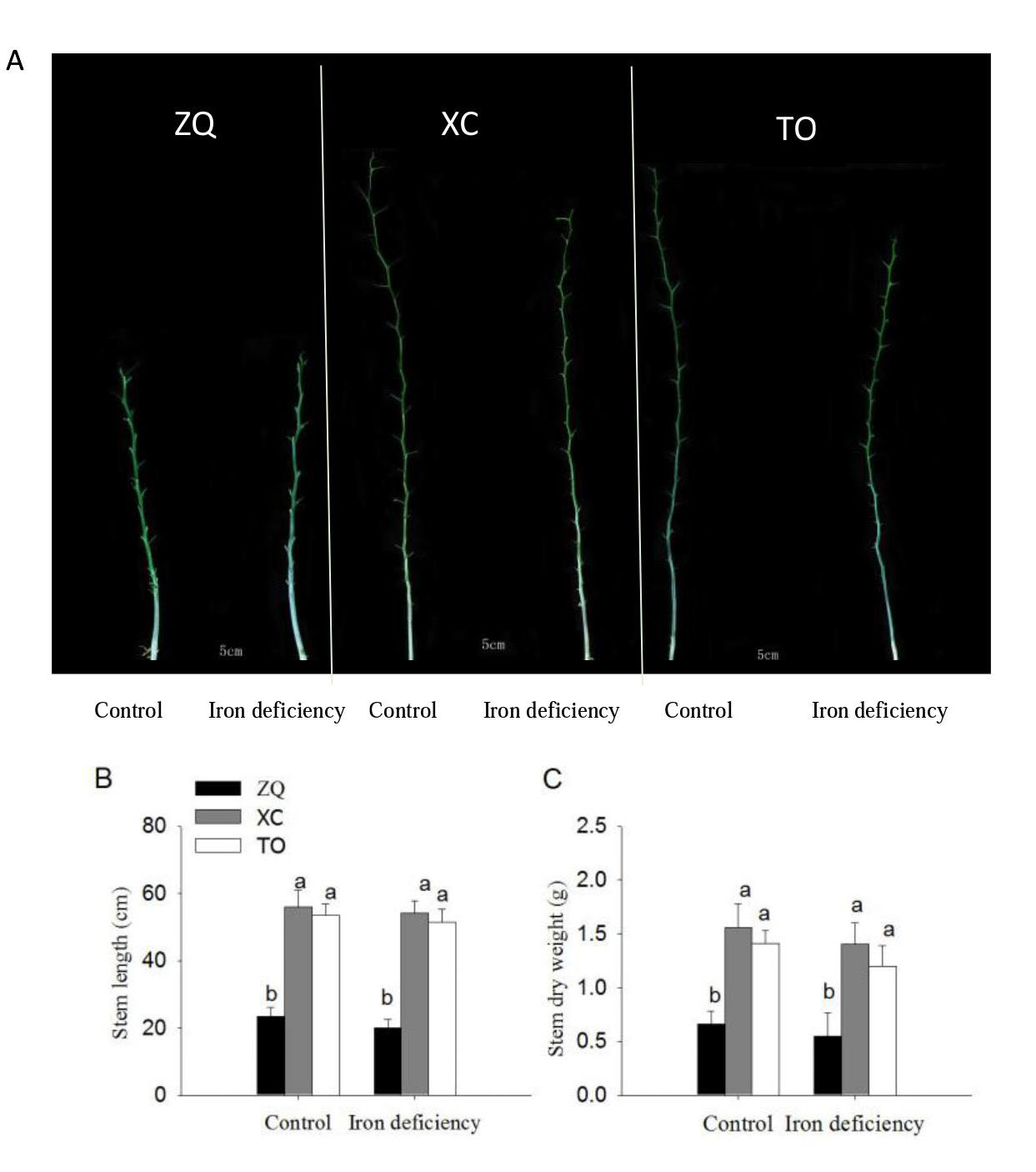

Supplement: FIGURE S1 — Stem growth parameters of Zhique (ZQ), Xiangcheng (XC), and trifoliate orange (TO) grown in control and iron deficiency nutrient solution for 100 days. (A) Stem morphology in ZQ, XC, and TO. (B) Stem length in ZQ, XC, and TO. (C) Stem dry weight in ZQ, XC, and TO. Letters (a, b, and c) indicate significant differences within samples via Duncan’s multiple range test P < 0.05 (Means ± SEM, n = 3). [file Image_1.JPEG]

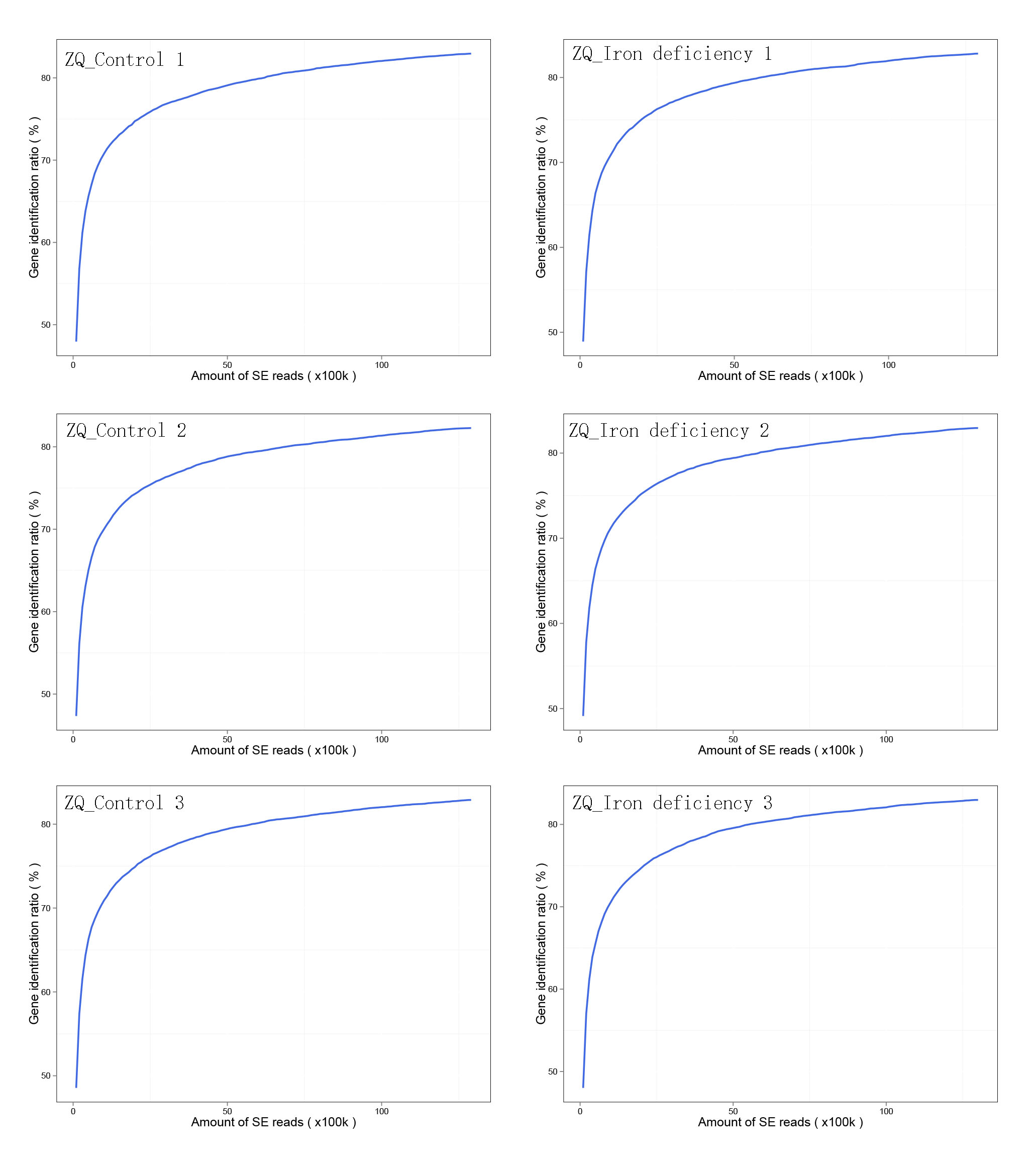

Supplement: FIGURE S2 — Curve of sequencing saturation in ZQ. X-axis shows the number of clean reads, units is 100 k – extreme value is currently the volume of sequencing. Y-axis shows the ratio of identified gene number to number of total gene reported in database. [file Image_2.jpg]

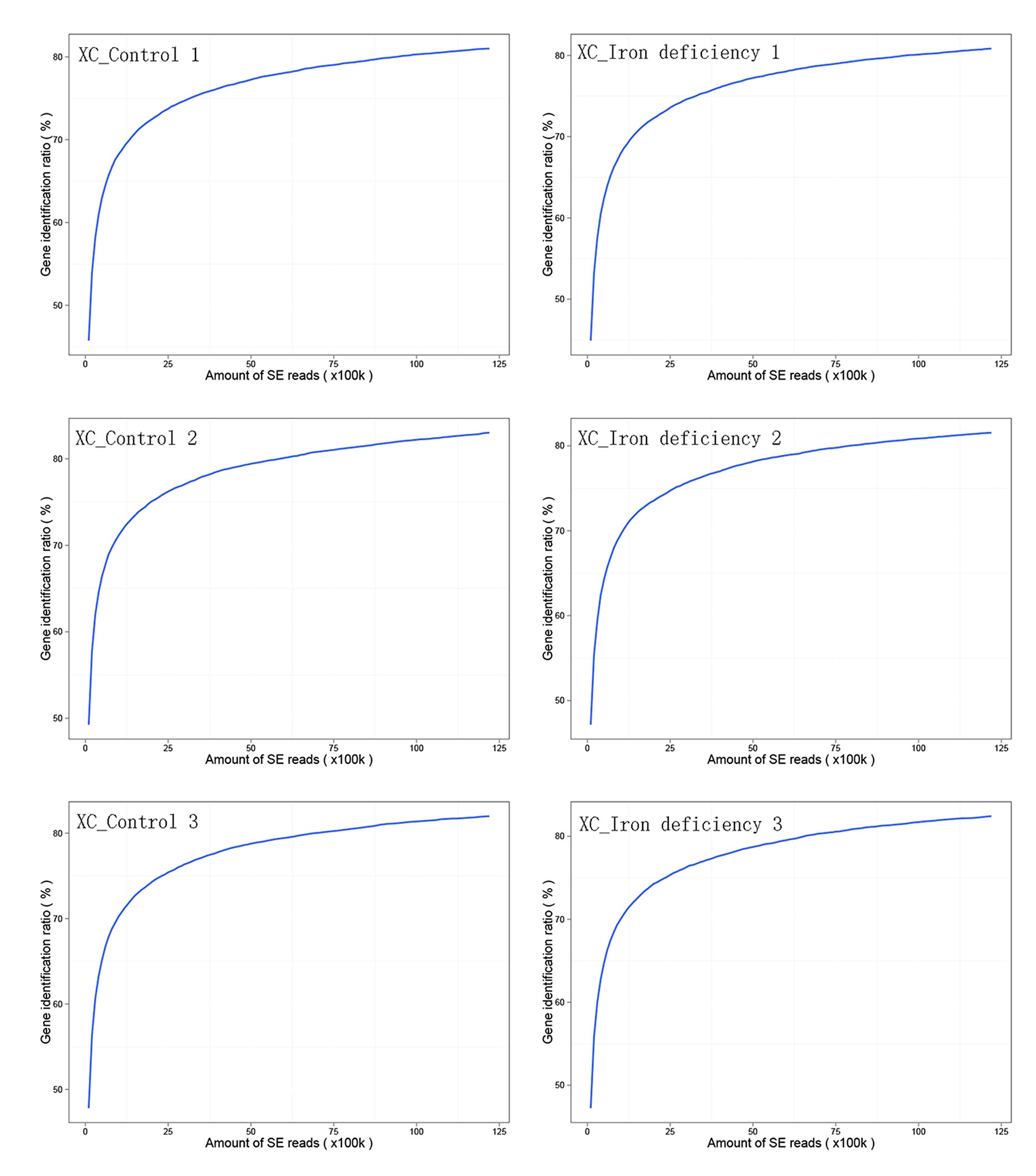

Supplement: FIGURE S3 — Curve of sequencing saturation in ZQ. X-axis shows the number of clean reads, units is 100 k – extreme value is currently the volume of sequencing. Y-axis shows the ratio of identified gene number to number of total gene reported in database. [file Image_3.jpg]

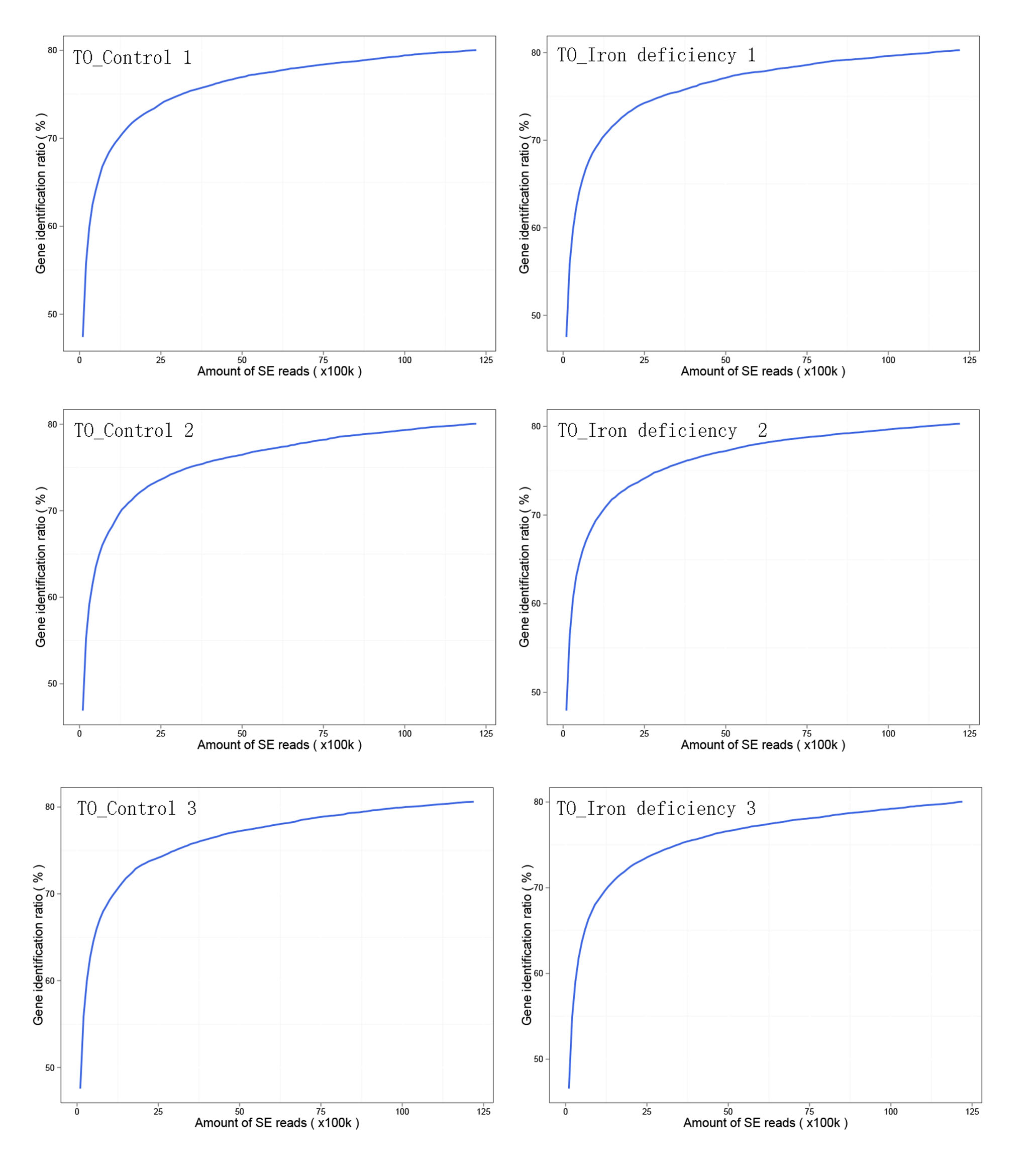

Supplement: FIGURE S4 — Curve of sequencing saturation in ZQ. X-axis shows the number of clean reads, units is 100 k – extreme value is currently the volume of sequencing. Y-axis shows the ratio of identified gene number to number of total gene reported in database. [file Image_4.jpg]

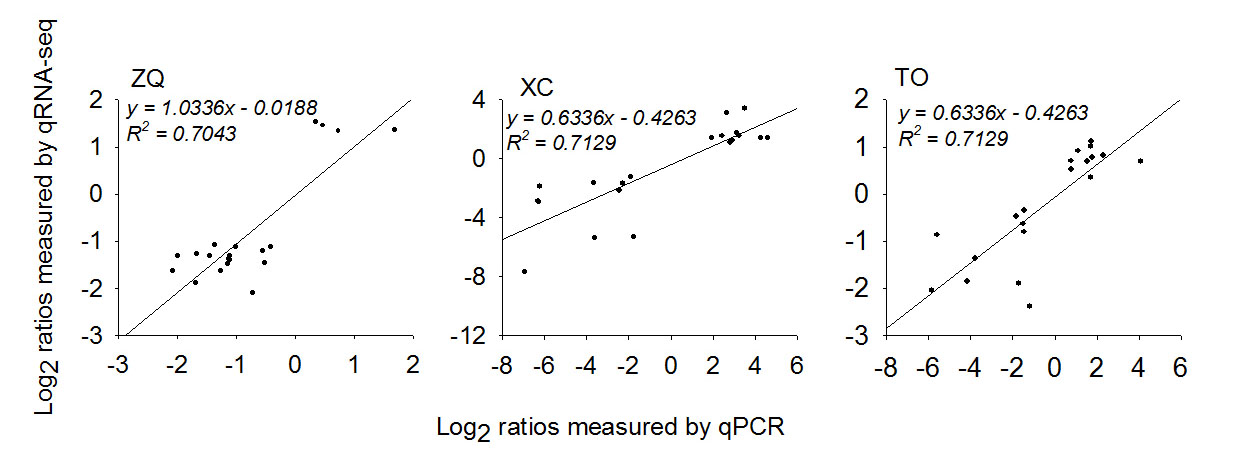

Supplement: FIGURE S5 — Log2 transcription levels under iron deficiency relative to control levels estimated by qRT-PCR and by RNA-seq for ZQ, XC, and TO after 24 h of iron deficiency. [file Image_5.JPEG]
